# Sedentary behavior and physical activity on exercise capacity in adult patients with congenital heart disease

**DOI:** 10.1016/j.ijcchd.2025.100569

**Published:** 2025-01-22

**Authors:** Masahiro Matsui, Keisei Kosaki, Naoto Kawamatsu, Yoshihiro Nozaki, Tomoko Machino-Otsuka, Yoshio Nakata, Seiji Maeda, Tomoko Ishizu

**Affiliations:** a Institute of Health and Sport Sciences, University of Tsukuba, Ibaraki, Japan; b Institute of Health and Sports Science & Medicine, Juntendo University, Chiba, Japan; c Department of Cardiology, Institute of Medicine, University of Tsukuba, Ibaraki, Japan; d Department of Child Health, Institute of Medicine, University of Tsukuba, Ibaraki, Japan; e Faculty of Sport Sciences, Waseda University, Saitama, Japan

**Keywords:** Daily behavior, Lifestyle, Accelerometer, Exercise capacity, Peak oxygen uptake

## Abstract

**Background:**

Reduced exercise capacity is associated with a poor prognosis in adult patients with congenital heart disease (CHD). Reducing sedentary behavior (SB) and increasing physical activity (PA) could be potential strategies that may contribute to enhanced fitness and prevention of acquired cardiovascular disease in adult patients with CHD. The present study aimed to examine the association of SB and PA with exercise capacity in adult patients with CHD.

**Methods:**

Ninety-six adult patients with CHD (age: 18–74 years) underwent measurements of peak oxygen uptake (VO_2_), % predicted peak VO_2_, and time spent in SB, light physical activity (LPA), and moderate-to-vigorous physical activity (MVPA). Three regression models (single-activity, partition, and isotemporal substitution) were used to examine the associations of the time spent in SB, LPA, and MVPA with peak VO_2_ and % predicted peak VO_2_.

**Results:**

In the single-activity and partition models, time spent in MVPA was consistently associated with peak VO_2_ and % predicted peak VO_2_. The isotemporal substitution model indicated that replacing 10-min of SB with the same duration of MVPA was associated with a higher peak VO_2_ (by 0.454 mL/min/kg [0.100 mL/min/kg, 0.807 mL/min/kg]) and % predicted peak VO_2_ (by 1.810 % [0.594 %, 3.026 %]).

**Conclusion:**

These findings suggest that reducing SB time and increasing PA time are associated with improved exercise capacity in adult patients with CHD.

## 1 Introduction

Congenital heart disease (CHD) occurs in approximately 1 % of all newborns. Improvements in cardiac management and surgical techniques have led to increased survival of patients with CHD, with over 90 % of infants reaching adulthood [1]. With these advances, the number of adult patients with CHD has continued to increase worldwide [[2], [3], [4], [5]]. However, recent studies have highlighted new problems in CHD patients such as age-related complications and the need for reinterventions [6,7]. Therefore, in addition to medication, lifestyle modifications are also important to extend the healthy life expectancy of adult patients with CHD.

Reduced exercise capacity is a common phenotype in patients with CHD. In a previous study that reviewed exercise capacity, peak oxygen uptake in adult patients with CHD was approximately 40%–80 % of that of the age- and sex-matched control group [8]. Additionally, a more pronounced reduction was closely related to a worse prognosis [[9], [10], [11]]. Accumulating evidence has shown that exercise training (cardiac rehabilitation) can improve exercise capacity in adult patients with CHD [12]. Japanese cardiac rehabilitation guidelines recommend performing exercise more than three times a week for patients with acquired cardiovascular disease [13]; however, since most adult patients with CHD are young or middle-aged, it is not feasible for them to participate in cardiac rehabilitation three times a week. Although long-term follow-up after the first repair is the main focus for many adult patients with CHD, cardiovascular rehabilitation is not covered by health insurance in Japan for patients without signs of heart failure, elevated brain natriuretic peptide (BNP) levels, or decreased exercise capacity. These aspects highlight the need to establish a strategy to increase exercise capacity that can be practiced in daily life.

In a scientific statement from the American Heart Association, a physically active lifestyle has been shown to be beneficial for improving health [14]. However, a previous study showed that the current recommendations for physical activity (PA) are not met in approximately half of the patients with CHD [15]. Despite the absence of evidence limiting recreational PA in most patients with CHD, such activity in this population is usually limited [16]. In addition, excessive sedentary behavior (SB) is unfavorable for cardiovascular health [17,18]. A recent review proposed that reducing SB and increasing PA are useful strategies for improving fitness and preventing acquired cardiovascular disease [19]. However, the relationship of the time spent in SB and PA with exercise capacity in adult patients with CHD has not been established. During awakening, daily activities are divided into SB, light physical activity (LPA), and moderate-to-vigorous physical activity (MVPA). Since the duration of a day is fixed and finite, to increase the time spent in one activity (e.g., MVPA), the time spent in other activities has to be reduced (e.g., SB). The present study aimed to examine the cross-sectional association of SB and PA with exercise capacity, considering this interdependence. We hypothesized that a shorter SB time and longer LPA and MVPA time are associated with better exercise capacity.

## 2 Methods

### 2.1 Participants

Adult patients with CHD who visited the University of Tsukuba Hospital were recruited for this study. A total of 96 adult patients with CHD (age, 18–74 years) completed the present examination. The study procedures were reviewed and approved by the Ethics Committee of the University of Tsukuba Hospital (approval number: R03-079) and conformed to the principles of the Declaration of Helsinki. Before participation, all the participants signed an informed consent form.

### 2.2 Sedentary behavior and physical activity

SB and PA were measured using a triaxial accelerometer (Active style Pro HJA-750C; Omron Healthcare Inc., Kyoto, Japan). This device can measure acceleration signals in triaxial directions (anteroposterior, mediolateral, and vertical) and estimate activity intensity using METs. These methods have been validated and used in previous studies [[20], [21], [22], [23]]. In this study, participants wore the device on their left waist for 7 days, except for the time spent sleeping and performing water-related activities (e.g., bathing and swimming). Acceleration signals were recorded every 10 s and accumulated every minute. To ensure data reliability, participants were required to wear the accelerometer for at least 10 h/day for at least three days (including one weekend day) [24]. The time spent in SB (≤1.5 METs), LPA (1.6–2.9 METs), and MVPA (≥3.0 METs) were calculated according to the estimated METs. Additionally, the average time spent in SB and PA of different intensity levels was calculated using the following formulas: (average weekday hours × 5) + (average holiday hours × 2)/7.

### 2.3 Exercise capacity

Cardiopulmonary exercise testing (CPX) was conducted on an electromagnetically braked cycle ergometer (Strength Ergo 8; Mitsubishi Electric Engineering Co., Ltd., Tokyo, Japan) in an upright position using a standard ramp protocol, as described previously [25]. Briefly, after a 4-min rest on the ergometer, the patients performed a 4-min warm-up at 0 W and 50 rpm. Following the warm-up, the ergometer load was increased by 1 W every 4, 6, or 12 s. Each patient's exercise load protocol was determined and implemented by a skilled cardiologist. Oxygen uptake was measured by using an online computer-assisted circuit spirometer (AE-300s; Minato Medical Science Co., Osaka, Japan). The average of the last 30 s of oxygen uptake during incremental exercise testing was defined as peak oxygen uptake (VO_2_). The % predicted peak VO_2_ was calculated using the following formula: % predicted peak VO_2_ = measured peak VO_2_/predicted peak VO_2_. Predicted peak VO_2_ was defined as the reference value for the normal Japanese population [26]. The minuet ventilation/carbon dioxide production (VE/VCO_2_) was calculated by linear regression fitting using VE and VCO_2_ obtained throughout the whole exercise [27].

### 2.4 Clinical measurements

A retrospective chart review was performed to obtain information on age, sex, height, weight, body mass index (BMI), cyanosis (saturation of percutaneous oxygen <94 %), and physician-assigned New York Heart Association (NYHA) classification (four categories ranging from class Ⅰ to class Ⅳ). Additionally, the CHD anatomical and physiological classification system was adopted as the physiological stage to classify the severity of CHD more comprehensively, including not only CHD anatomy but also the presence of complicated valvular disease, cyanosis, pulmonary hypertension, heart failure symptoms, and complicated arrhythmia. According to the 2018 AHA/ACC Guideline for the Management of Adults With Congenital Heart Disease, the CHD anatomy was classified as simple, moderate complexity, or great complexity, and the physiological stage was graded as A, B, C, or D [28].

### 2.5 Statistical analysis

Three linear regression models (single-activity, partition, and isotemporal substitution) were used to examine the associations of time spent in SB, LPA, and MVPA with exercise capacity. For all analyses, the time spent in SB, LPA, and MVPA was divided by 10-min units to estimate the effect of short-term behavioral changes on exercise capacity. Thus, the results were interpreted as the change in peak VO_2_ or % predicted peak VO_2_ when each activity was varied for 10 min. This time unit was decided according to the current PA guideline from the American College of Sports Medicine and the American Heart Association [29] and the recommendation in the official Japanese PA guidelines that an additional 10 min/day of PA contributes to a long healthy life expectancy [30]. All statistical models were adjusted for potential covariates such as age and sex (if the association with peak VO_2_ was examined), BMI, CHD anatomy (simple, moderate, or complex), and physiological stage (A·B or C·D). Additionally, non-standardized regression coefficients for each activity corresponded to the effect of a 10-min increase in peak VO_2_ or % predicted peak VO_2_.

The single-activity model assessed the association between each activity (SB, LPA, or MVPA) and exercise capacity, considering the total wear time and covariates. In this model, the dependent variable was peak VO_2_ or % predicted peak VO_2_, and the independent variables were the time spent in each activity, accelerometer wear time, and covariates. The partition model estimated the change in exercise capacity based on the time spent engaging in each activity when holding the time spent in other activities constant and controlling for covariates. In this model, the dependent variable was peak VO_2_ or % predicted peak VO_2_, and the independent variables were time spent in all activities (SB, LPA, and MVPA) and covariates, but not accelerometer wear time.

The effect of replacing the time spent in one activity (e.g., SB) with the same time spent in other activities (e.g., MVPA) on exercise capacity was estimated using the isotemporal substitution model. When SB was replaced with other activities, the isotemporal substitution model was expressed as follows: exercise capacity = (b1) LPA + (b2) MVPA + (b3) accelerometer wear time + (b4) covariates. The coefficients b1 and b2 in this model indicate the effect of replacing 10 min of SB with LPA (b1) or MVPA (b2) while holding the other activity (i.e., MVPA or LPA) and accelerometer wear time constant.

Two-way analysis of covariance was performed to examine the joint association between the MVPA time divided into two groups by the median and CHD anatomy or physiological stage on peak VO_2_ or % predicted peak VO_2_. These associations were adjusted for BMI. The % predicted peak VO_2_ was considered by age and sex. Therefore, age and sex were adjusted only when examining the associations for peak VO_2_.

All data are described as mean ± standard deviation or number (%) unless stated otherwise. All statistical analyses were performed using SPSS Statistics version 28.0 (IBM Japan, Tokyo, Japan). Statistical significance was defined as p < 0.05.

## 3 Results

Table 1 shows the characteristics of 96 adult patients with CHD. The mean age of the patients was 36 ± 15 years, and the percentage of women was 50 % (n = 48). On average, the time spent in SB, LPA, and MVPA was 565 ± 130, 332 ± 117, and 50 ± 32 min/day, respectively. The mean values of the peak VO_2_ and % predicted peak VO_2_ were 21.7 ± 6.3 mL/min/kg and 77 % ± 19 %.

**Table 1:** Participant characteristics.

n	96
Age, years	36 ± 15
Women, n (%)	48 (50)
CHD anatomy	
Simple, n (%)	20 (21)
Moderate complexity, n (%)	41 (43)
Great complexity, n (%)	35 (37)
Physiological stage	
Stage A, n (%)	6 (6)
Stage B, n (%)	28 (29)
Stage C, n (%)	56 (58)
Stage D, n (%)	6 (6)
NYHA class	
Class Ⅰ, n (%)	44 (46)
Class Ⅱ, n (%)	39 (41)
Class Ⅲ, n (%)	13 (14)
Cyanosis, n (%)	9 (9)
Height, cm	162 ± 8
Weight, kg	58 ± 13
Body mass index, kg/m^2^	22.1 ± 3.9
Peak VO_2_, mL/min/kg	21.7 ± 6.3
% predicted peak VO_2_, %	77 ± 19
VE/VCO_2_ slope ≥34, n (%)	21 (22)
Accelerometer wear time, min/day	946 ± 79
SB time, min/day	565 ± 130
LPA time, min/day	332 ± 117
MVPA time, min/day	50 ± 32

Data are expressed as mean ± standard deviation.

Abbreviations: CHD, congenital heart disease; SB, sedentary behavior; LPA, light-intensity physical activity; MVPA, moderate-to vigorous-intensity physical activity.

Table 2 presents the results of the three regression models for peak VO_2_. The single-activity model indicated that the time spent in SB, LPA, and MVPA were associated with peak VO_2_ (all tests, p < 0.05). The partition model showed an association between the time spent in LPA and MVPA and peak VO_2_ (all tests, p < 0.05). The isotemporal substitution model showed that replacing 10 min of SB with the same duration of LPA or MVPA was significantly associated with a higher peak VO_2_ (all tests, p < 0.05). However, replacing 10 min of LPA with MVPA was not associated with a peak VO_2_ (p = 0.125).

**Table 2:** Multivariable regression analysis for peak VO_2_.

	SB time	LPA time	MVPA time
	B (95 % CIs)	B (95 % CIs)	B (95 % CIs)
Single-activity model	−0.185 (−0.264, −0.106) ∗	0.192 (0.100, 0.285) ∗	0.664 (0.334, 0.993) ∗
Partition model	0.009 (−0.122, 0.141)	0.146 (0.004, 0.287) ∗	0.463 (0.093, 0.833) ∗
Isotemporal substitution model			
Replacing SB time	Dropped	0.137 (0.037, 0.237) ∗	0.454 (0.100, 0.807) ∗
Replacing LPA time	−0.137 (−0.237, −0.037) ∗	Dropped	0.317 (−0.090, 0.724)
Replacing MVPA time	−0.454 (−0.807, −0.100) ∗	−0.317 (−0.724, 0.090)	Dropped

Data are expressed as non-standardized regression coefficient (95 % confidence intervals).

∗: p < 0.05.

Abbreviations: SB, sedentary behavior; LPA, light physical activity; MVPA, moderate-to-vigorous physical activity.

The results of the three regression models for the % predicted peak VO_2_ are presented in Table 3. The single-activity models showed associations between the time spent in SB, LPA, and MVPA and the % predicted peak VO_2_ (all tests, p < 0.05). The partition model showed that only the time spent in MVPA was associated with the % predicted peak VO_2_ (p < 0.05), but not with the SB and LPA time. In the isotemporal substitution model, replacing 10 min of SB or LPA with the same time of MVPA was associated with a higher % predicted peak VO_2_ (all tests, p < 0.05).

**Table 3:** Multivariable regression analysis for % predicted peak VO_2_.

	SB time	LPA time	MVPA time
	B (95 % CIs)	B (95 % CIs)	B (95 % CIs)
Single-activity model	−0.505 (−0.787, −0.223) ∗	0.487 (0.155, 0.819) ∗	2.199 (1.088, 3.310) ∗
Partition model	0.016 (−0.461, 0.493)	0.283 (−0.217, 0.782)	1.826 (0.535, 3.117) ∗
Isotemporal substitution model			
Replacing SB time	Dropped	0.267 (−0.085, 0.618)	1.810 (0.594, 3.026) ∗
Replacing LPA time	−0.267 (−0.618, 0.085)	Dropped	1.543 (0.143, 2.944) ∗
Replacing MVPA time	−1.810 (−3.026, −0.594) ∗	−1.543 (−2.944, −0.143) ∗	Dropped

Data are expressed as non-standardized regression coefficient (95 % confidence intervals).

∗: p < 0.05.

Abbreviations: SB, sedentary behavior; LPA, light physical activity; MVPA, moderate-to-vigorous physical activity.

**Fig. 1:**
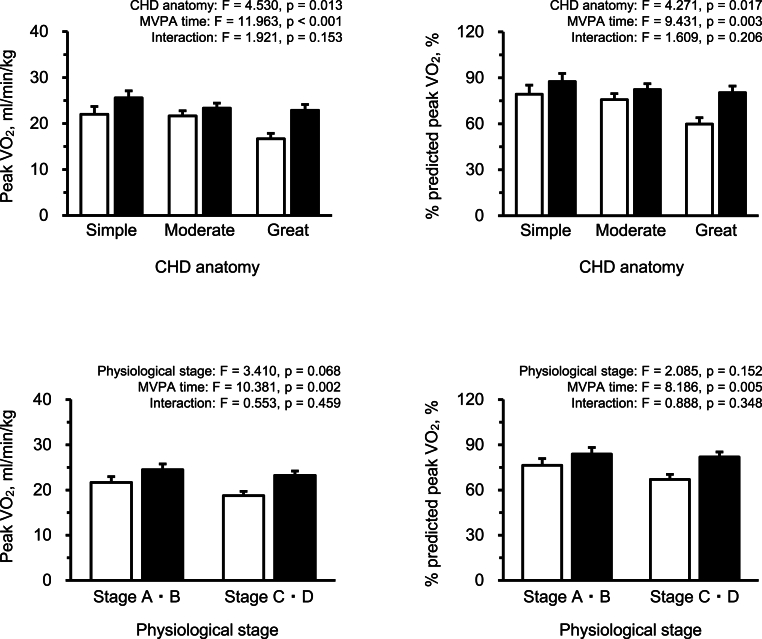
The joint associations of CHD anatomy or physiological stage and MVPA time with peak VO_2_ and % predicted peak VO_2_. Data are expressed as mean ± standard error. White and black bars indicate individuals with lower and higher MVPA levels, respectively. These associations were adjusted for body mass index. Age and sex were adjusted only when examining the associations for peak VO_2_, but not % predicted peak VO_2_. Statistical values were evaluated using a two-way analysis of covariance. Abbreviations: CHD, congenital heart disease; MVPA, moderate-to-vigorous physical activity.

Fig. 1 shows the joint associations of CHD anatomy or physiological stage and MVPA time with peak VO_2_ and % predicted peak VO_2_. The main effects of MVPA time on peak VO_2_ and % predicted peak VO_2_ were significant in all models (all tests, p < 0.05). The main effects of CHD anatomy on peak VO_2_ and % predicted peak VO_2_ were also significant (all tests, p < 0.05). However, no interaction effect was observed between CHD anatomy or physiological stage and MVPA time on peak VO_2_ and % predicted peak VO_2_.

## 4 Discussion

This study examined the associations of daily SB, LPA, and MVPA time with exercise capacity. We found that the time spent in SB, LPA, and MVPA were respectively associated with peak VO_2_ and % predicted peak VO_2_ in the single-activity models. The partition model showed that MVPA time was consistently associated with peak VO_2_ and % predicted peak VO_2_. Additionally, in the isotemporal substitution models, favorable replacement effects on peak VO_2_ and % predicted peak VO_2_ were observed when 10 min SB time was replaced with the same amount of MVPA time. These findings suggest that daily behavior was associated with exercise capacity, and reducing SB time and increasing MVPA time may be important for maintaining or improving exercise capacity in adult patients with CHD. Moreover, our findings showed no interaction between CHD anatomy or physiological stage and MVPA time on exercise capacity. Thus, daily MVPA may be associated with maintained or improved exercise capacity regardless of disease severity.

The favorable effects of PA on health outcomes have been established in many populations, including those with acquired cardiovascular disease [19,31]. However, a previous study reported that adult patients with CHD were less active than healthy peers [32], and approximately half of them do not meet the current PA guidelines [15]. In addition to showing lower levels of PA, adult patients with CHD have poorer exercise capacity [8], which strongly predicts a poor prognosis [9,10]. These findings suggest that in addition to congenital heart defects, reduced daily PA (sedentary lifestyle) leads to decreased exercise capacity. Regarding the promotion of PA, a scientific statement from the American Heart Association recommends low-risk PA tailored to each congenital heart defect and the patient's medical condition [14]. Thus, PA need not be completely limited, and low-risk PA may be encouraged in these patients. In this study, we demonstrated that a shorter SB time and longer PA time were associated with higher levels of peak VO_2_, and that replacing SB time with the same time spent in MVPA was associated with higher peak VO_2_. Favorable lifestyle (i.e., shorter SB time and longer PA time) may be associated with improved exercise capacity and later prognosis in adult patients with CHD.

Previous studies have reported that exercise training (cardiac rehabilitation) in hospitals can improve the peak VO_2_ in patients with CHD [12]. Importantly, no adverse events were identified with the exercise interventions, underscoring the potential of exercise in improving exercise capacity in patients with CHD. However, continuous participation in cardiac rehabilitation may be difficult for largely employed patients with CHD. A previous study examining patients with heart failure in Japan showed that the rate of cardiac rehabilitation participation among outpatients was only 7 % [33]. This suggests that the percentage of participation in cardiac rehabilitation is low among patients with CHD. Moreover, although long-term follow-up after the first repair is the main focus for many adult patients with CHD, cardiovascular rehabilitation is not covered by health insurance in Japan for patients without signs of heart failure, elevated BNP levels, or decreased exercise capacity. These findings highlight a need to move the intervention setting from the hospital to the home. In the present study, peak VO_2_ showed higher values when SB time in daily life was replaced by PA time. These results are consistent with the findings of previous studies on supervised and home-based exercise interventions in patients with CHD [12,34]. Modification of daily life behaviors may be a new intervention method for many patients with CHD.

The maintenance and improvement of exercise capacity are closely related to a lower risk of death and other adverse outcomes. Diller et al. showed that a unit increase in peak VO_2_ (mL/min/kg) predicted a lower risk of hospitalization or death (hazard ratio [95 % CI], 0.937 [0.890, 0.986]) in adult patients with CHD [9]. Additionally, a unit increase in % predicted peak VO_2_ (%) was related to a lower risk of death or sustained ventricular tachycardia (hazard ratio [95 % CI]: 0.968 [0.948, 0.988]) in patients late after surgical repair of tetralogy [35]. In the present study, replacing 10 min of SB per day with the same amount of MVPA resulted in a higher peak VO_2_ (by 0.454 mL/min/kg [0.100 mL/min/kg, 0.807 mL/min/kg]) and % predicted peak VO_2_ (by 1.810 % [0.594 %, 3.026 %]). These results suggest that daily activity plays an important role in exercise capacity and that advising CHD patients to replace daily SB time with PA time may contribute to improving the prognosis of adult patients with CHD.

This study showed no interaction effects of the time spent in MVPA and CHD anatomy or physiological stage on exercise capacity, which suggests that PA recommendations might not need to differ based on these variables alone. Nevertheless, given that the severity of CHD varies greatly among individuals, a blanket recommendation for daily PA may not be appropriate for all CHD patients. Although PA is not generally linked with increased CHD-related adverse events [12], it is especially crucial to consider that clinical reports highlight an association between sudden cardiac deaths and exercise or active physical exertion, particularly in patients with transposition of the great arteries [36]. Therefore, while promoting PA for its potential benefits, it is essential to tailor recommendations to individual risk profiles and to conduct further research to ascertain the safety and efficacy of PA across the spectrum of adult patients with CHD.

The present study had several limitations. First, because this was a cross-sectional examination with a small sample recruited from a single center, the generalizability of these findings to all adult patients with CHD is unclear. In addition, causal and long-term relationships of time spent in SB and PA with exercise capacity could not be determined. Further prospective studies should be conducted to clarify these causal and long-term relationships. Next, although the present study showed a correlation of the time spent in SB and PA with exercise capacity, these relationships may vary depending on the environment surrounding the patients’ PA and fitness track from childhood to adulthood [37]. The diagnosis of CHD during childhood results in patient overprotection. Overprotection by the surrounding adults may lead to an inactive lifestyle and low physical fitness [38,39]. Therefore, future studies should examine whether the environment surrounding patients can moderate the association of the time spent in SB and PA with exercise capacity.

In conclusion, the present study indicated that the time spent in SB, LPA, and MVPA were respectively associated with peak VO_2_ and the % predicted peak VO_2_. Additionally, favorable replacement effects on peak VO_2_ and % predicted peak VO_2_ were observed when SB was replaced with the same time of MVPA. These findings suggest that reducing SB and increasing PA time are associated with improved exercise capacity in adult patients with CHD.

## CRediT authorship contribution statement

**Masahiro Matsui:** Writing – original draft, Investigation, Formal analysis. **Keisei Kosaki:** Writing – review & editing, Funding acquisition. **Naoto Kawamatsu:** Writing – review & editing, Investigation, Funding acquisition. **Yoshihiro Nozaki:** Writing – review & editing. **Tomoko Machino-Otsuka:** Writing – review & editing, Investigation. **Yoshio Nakata:** Writing – review & editing. **Seiji Maeda:** Writing – review & editing. **Tomoko Ishizu:** Writing – review & editing, Project administration, Investigation, Conceptualization.

## Data availability

The data that support the findings of this study are available on request from the corresponding author. The data are not publicly available due to privacy or ethical restrictions.

## Declaration of competing interest

The authors declare that they have no known competing financial interests or personal relationships that could have appeared to influence the work reported in this paper.
